# β-Cyclodextrin-Modified Laser-Induced Graphene Electrode for Detection of *N*6-Methyladenosine in RNA

**DOI:** 10.3390/molecules29194718

**Published:** 2024-10-05

**Authors:** Jingyi Guo, Mei Zhao, Xia Kuang, Zilin Chen, Fang Wang

**Affiliations:** Key Laboratory of Combinatorial Biosynthesis and Drug Discovery (MOE), School of Pharmaceutical Sciences, Wuhan University, Wuhan 430071, China; 2022203060028@whu.edu.cn (J.G.);

**Keywords:** electrochemical biosensor, *N*6-methyladenosine, laser-induced graphene, nucleic acid methylation

## Abstract

Laser-induced graphene (LIG) possesses characteristics of easy handling, miniaturization, and unique electrical properties. We modified the surface of LIG by electropolymerizing β-cyclodextrin (β-CD), which was used to immobilize antibodies on the electrode surface for highly sensitive detection of targets. *N*6-methyladenosine (m6A) is the most prevalent reversible modification in mammalian messenger RNA and noncoding RNA, influencing the development of various cancers. Here, β-CD was electropolymerized to immobilize the anti-m6A antibody, which subsequently recognized the target m6A. This was integrated into the catalytic hydrogen peroxide–hydroquinone (H_2_O_2_-HQ) redox system using phos-tag-biotin to generate electrochemical signals from streptavidin-modified horseradish peroxidase (SA-HRP). Under optimal conditions, the biosensor exhibited a linear range from 0.1 to 100 nM with a minimum detection limit of 96 pM. The method was successfully applied to the recovery analysis of m6A from HeLa cells through spiking experiments and aims to inspire strategies for point-of-care testing (POCT).

## 1. Introduction

Since its discovery in 2014 [1], laser-induced graphene (LIG) has emerged as a versatile material for a variety of applications, including sensors [2,3,4], supercapacitors [5,6,7], catalysts [8,9], and batteries [10,11]. This versatility stems from LIG’s ease of graphical design and preparation, ability to be miniaturized, simplicity in manipulation, large effective surface area, and excellent electrical conductivity. Specifically, in the realm of electrochemical sensors, LIG has facilitated the detection of diverse molecules such as nitrite [12,13], dopamine [14], glucose [15,16,17], disease biomarkers [18], and miRNA [19], highlighting its potential as a substrate electrode for point-of-care testing (POCT) [18,20]. In addition, the use of electrochemically active materials is crucial for the effective operation of electrochemical biosensors [21]. LIG-based biosensors can also monitor environmental pollutants, facilitating the ready and most local detection of pollutants to avoid human health threats and environmental hazards. Meanwhile, LIG biosensors have a lot of room for development for detecting biomarkers such as DNA, RNA, proteins, peptides, and cells [22]. The feasibility of LIG-based fabricated electrochemical biosensors being used for biomolecule detection is demonstrated.

RNA methylation, an important component of epigenetic studies, refers to the methylation modification occurring at various positions on RNA molecules [23]. N6-methyladenosine (m6A) is the most prevalent and reversible internal modification of mammalian messenger RNA and noncoding RNA. In mammals, m6A modifications affect many aspects of RNA metabolism [24], including mRNA stability [25,26], alternative splicing [27,28], translation [29,30], gene expression regulation [31], and various other biological processes. It has been shown that abnormal m6A levels are associated with the development of multiple cancers [23,32,33,34,35]. Therefore, it is crucial to develop methods for real-time quantitative detection of m6A and its related enzyme levels. Currently, several common methods for m6A modification detection are utilized, such as methylated RNA immunoprecipitation with next-generation sequencing (MeRIP-seq) [36], the liquid chromatography tandem mass spectrometry technique (LC-MS/MS) [37,38], and the individual nucleotide resolution cross-linking and immunoprecipitation (miCLIP) [39] technique, which provide effective approaches for m6A detection, but the large-scale equipment and higher cost of operation may limit the general application of these methods. Electrochemical methods have also been used for the detection of m6A due to their advantages of high sensitivity, good selectivity, time-saving, and not needing large-scale instruments [40,41,42,43,44,45]. For example, Yin et al. immobilized the antibody using the specific interaction of 4-aminophenylboronic acid (APBA) with the sugar moiety of the m6A antibody and realized the detection of m6A based on the decrease in electrochemical signals obtained by electrostatic repulsion of [Fe(CN)_6_]^3−^ with the electrode and applied it to the study of phytohormone effects on the level of m6A in the leaves of rice seedlings [46]. In addition, Ai’s team utilized phos-tag-biotin to link m6ATP and Ag@SiO_2_, providing a promising strategy for the detection of ribonucleotides and deoxyribonucleotides [47]. Ou et al. synthesized an m6A-DNA-PtCo-type probe, which competed with m6A for the antibody on a gold electrode used as a signal probe [48]. The method exhibits rapid detection capabilities alongside an expansive linear range, presenting an innovative approach for the accurate quantification of m6A.

In the current study, we harness these inherent benefits of LIG to develop an electrochemical biosensor tailored for the detection of m6A. This is achieved by first graphically designing LIG electrodes, which are then modified by the electropolymerization of β-cyclodextrin (β-CD) onto the LIG surface. The immobilization of the m6A-specific antibody onto the β-CD/LIG matrix through noncovalent bonds enables the selective recognition of m6A. To mitigate nonspecific adsorption, bovine serum albumin (BSA) is applied to occlude any unbound m6A sites. Subsequently, phos-tag-biotin is employed to recognize the m6A chain’s phosphate group [49]. The culmination of this process involves the specific interaction between streptavidin and biotin, facilitating the hydrogen peroxide–hydroquinone (H_2_O_2_-HQ) redox reaction. As the concentration of bound m6A increases, so does the resultant current signal, allowing for the quantification of m6A. This methodical approach not only leverages the advantageous properties of LIG but also introduces a logical and causally related sequence of steps for effective m6A detection.

## 2. Results and Discussions

### 2.1. Characterization of Bare LIG and β-CD/LIG

**Figure 1:**
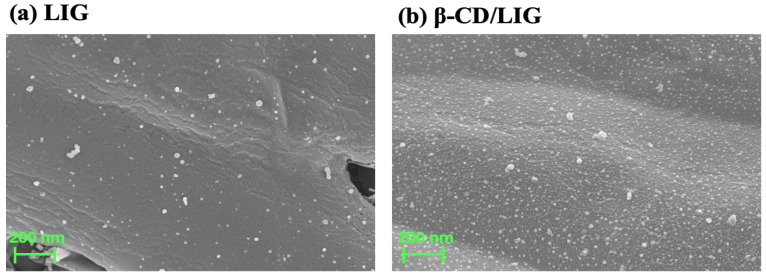
Scanning electron microscope (SEM) images of (**a**) LIG and (**b**) β-CD/LIG.

Figure 1 shows the scanning electron microscope (SEM) morphology of the LIG and β-CD/LIG electrodes. As shown in Figure 1a, it can first be observed that the LIG surface exhibits a 3D porous microstructure composed of alternating grooves and raised bars. This structure may result from the relatively poor precision of the semiconductor laser engraver after irradiation by the semiconductor laser source. Furthermore, the LIG electrode presents a 3D crumpled-like porous structure, which may be caused by the depolymerization–carbonization–graphitization process of PI film and leads to the graphitic product. Under the high-temperature conditions generated by the laser, the graphitization process may simultaneously produce oxygen and water, both of which can easily react with graphitic carbon to form carbon monoxide and carbon dioxide [16]. In Figure 1b, which displays the SEM images of β-CD/LIG, the growth of β-CD can be clearly observed, indicating the successful electropolymerization of β-CD.

**Figure 2:**
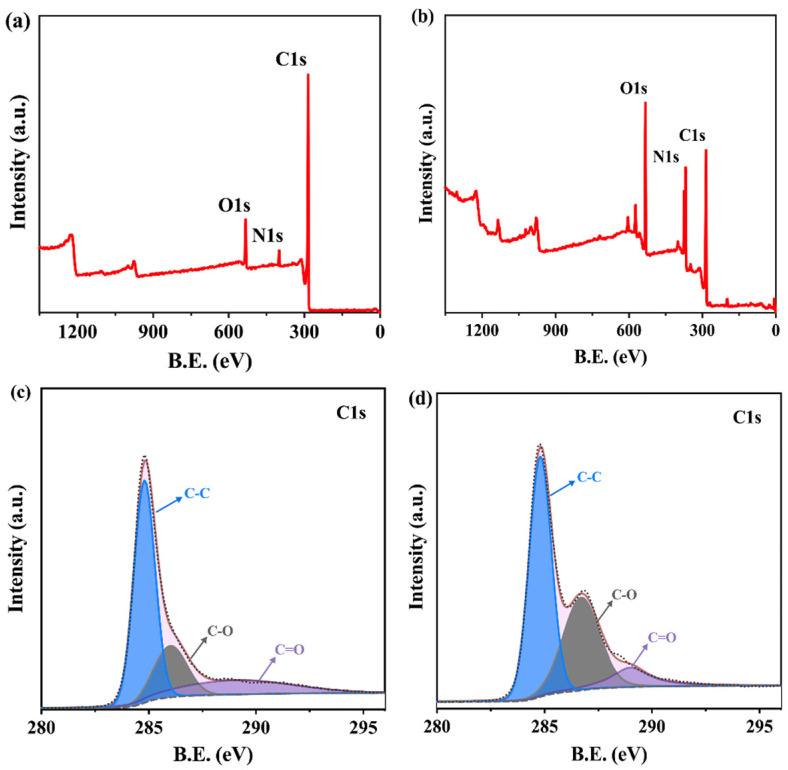
(**a**,**b**) show the full-scan X-ray photoelectron spectroscopy (XPS) spectrums of LIG and β-CD/LIG, respectively. (**c**,**d**) show the high-resolution C1s spectrums of LIG and β-CD/LIG, respectively.

X-ray photoelectron spectroscopy (XPS) was utilized to provide the chemical composition of the LIG and β-CD/LIG surfaces. The full-scan XPS spectra of LIG and β-CD/LIG are shown in Figure 2a,b, with the atomic percentages of C, N, and O species in LIG and β-CD/LIG samples supplied in the Supplementary Materials (Table S1). The composition of C, N, and O in the LIG samples was 88.32%, 3.89%, and 7.79%, respectively, validating the successful fabrication of graphene with relatively low oxidation. After the electropolymerization of β-CD on LIG, the atomic ratio of oxygen increased from 7.79% to 21.97%, which represents the increasing amounts of immobilized β-CD and implied the successful electropolymerization of β-CD. The high-resolution XPS spectrum of the C1s peak showed three characteristic peaks for LIG (Figure 2c) at 284.8 eV, 286.0 eV, and 289.1 eV, corresponding to C-C, C-O, and C=O, respectively [50]. For the β-CD/LIG, an obviously increasing proportion of the C-O characteristic peak (Figure 2d) at ~286.7 eV also indicated the electropolymerization of β-CD.

### 2.2. Electrochemical Characterization

**Figure 3:**
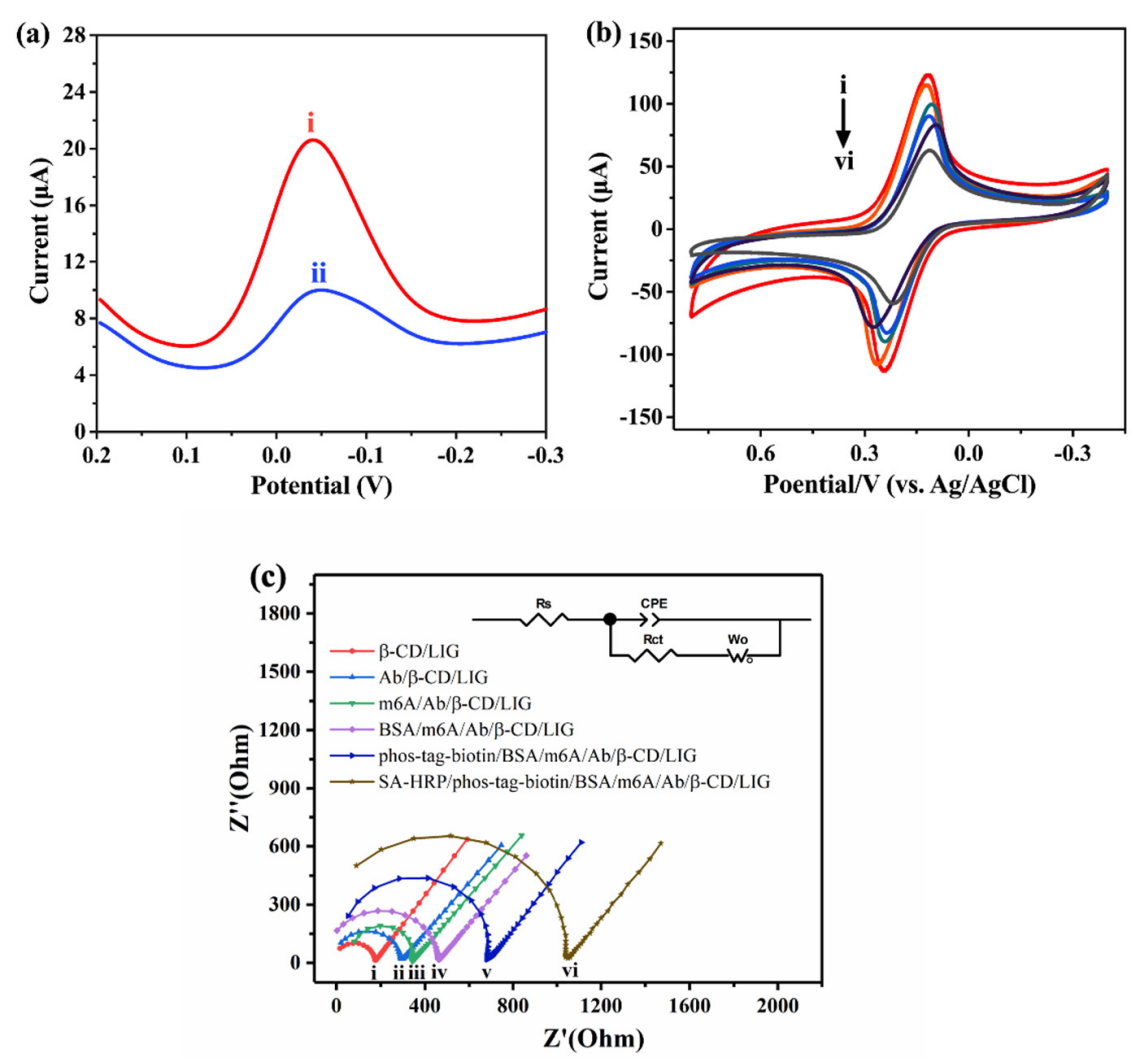
(**a**) Feasibility of the method at 0.01 µM m6A. i–ii: m6A, unmethylated A (control), and m6A–RNA. (**b**) Cyclic voltammetry plots (CVs) of the SA-HRP/phos-tag-biotin/BSA/m6A/Ab/β-CD/LIG at 0.01 µM m6A in 0.1 M KCl containing 2.5 mM K_3_[Fe(CN)_6_] and 2.5 mM K_4_[Fe(CN)_6_]. (**c**) Nyquist plots of SA-HRP/phos-tag-biotin/BSA/m6A/Ab/β-CD/LIG. i–vi: β-CD/LIG, Ab/β-CD/LIG, m6A/Ab/β-CD/LIG, BSA/m6A/Ab/β-CD/LIG, phos-tag-biotin/BSA/m6A/Ab/β-CD/LIG, and SA-HRP/phos-tag-biotin/BSA/m6A/Ab/β-CD/LIG, respectively. EIS frequency range: 0.1–400000 Hz, amplitude of 1000 µA.

Figure 3a shows the feasibility of this method. Compared to m6A (curve i), unmethylated A (curve ii) depicts a weaker peak current, which is due to the inability of unmethylated A to bind to the antibody. As a result, the complex cannot form with phos-tag-biotin and SA-HRP, leading to a weaker current. To verify the method’s feasibility, we investigated the electrochemical properties of the prepared LIG electrodes using cyclic voltammetry (CV) and electrochemical impedance spectroscopy (EIS).

Figure 3b depicts the CV plots of the layer-by-layer assembled electrodes in the [Fe(CN)_6_]^3−/4−^ redox system. The peak current (I_p_) of the β-CD/LIG (curve i) was the highest. Following the immobilization of the antibody, m6A, BSA, phos-tag-biotin, and SA-HRP (curves ii–vi), a stepwise decrease in I_p_ was observed step by step, which confirmed the successful binding of these molecules on β-CD/LIG. The effective surface area of the assembled electrodes could be calculated under the Randles–Sevcik equation [51]. The value of the surface area (Table S2) increased after electropolymerization of β-CD on the LIG electrode. The introduction of antibody, m6A, BSA, phos-tag-biotin, and SA-HRP hindered the electron transfer between [Fe(CN)_6_]^3−/4−^ and the electrode surface, thereby reducing the surface area. These results further verified the successful assembly process of the method. Where I_p_ is the peak current, A is the electrode area, n is the electron transfer number, D_0_ is the diffusion coefficient, and v is the scan rate.

(1) $$I_{p}=2.69\times 10^{5}\;A\;n^{3/2}\;{D_{0}}^{1/2}\;C_{0}\;v^{1/2}$$

Additionally, electrochemical impedance spectroscopy (EIS) measurements characterized the stepwise processes in a 0.1 M KCl solution containing 2.5 mM [Fe(CN)_6_]^3−^ and 2.5 mM [Fe(CN)_6_]^4−^, with Nyquist plots presented in Figure 3c. In detail, the Nyquist plots were defined with capacitance (CPE), resistances (Ret and Rs), and Warburg impedance (W). Compared with the β-CD/LIG electrode (curve i), after each modification of antibody, m6A, BSA, phos-tag-biotin, and SA-HRP (curve ii–vi), the impedance of the electrode subsequently became larger, which meant that the electron transfer process slowed down step by step. The EIS results are consistent with the CV results shown in Figure 3b, further demonstrating the successful assembly of the electrodes at each step of modification.

### 2.3. Optimization of Experimental Conditions

**Figure 4:**
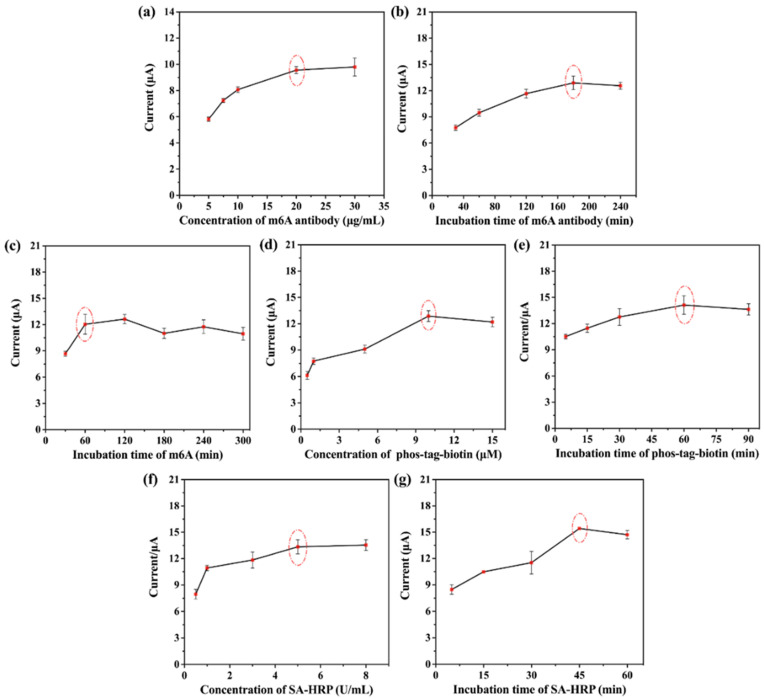
Optimization of experimental conditions. (**a**,**b**) Effect of m6A antibody concentration and incubation time at 0.01 µM m6A, respectively. (**c**) Effect of m6A incubation time at 0.01 µM m6A. (**d**,**e**) Effect of phos-tag-biotin concentration and incubation time at 0.01 µM m6A, respectively. (**f**,**g**) Effect of SA-HRP concentration and incubation time at 0.01 µM m6A, respectively. Error bars represent standard deviations of three measurements.

Several factors were tested to obtain the optimal experimental conditions, including m6A antibody concentration, m6A antibody incubation time, m6A incubation time, phos-tag-biotin concentration, phos-tag-biotin incubation time, SA-HRP concentration, and SA-HRP incubation time using the DPV technique. As shown in Figure 4a,b, we first explored the m6A antibody concentration and incubation time after β-CD electropolymerization. The current response gradually increased with the increasing in m6A antibody concentration and incubation time, stabilizing under certain conditions, which may be caused by the saturation of subject–object interaction between antibody and β-CD. The specific binding of m6A to the antibody was optimized at 1 h (Figure 4c). Figure 4d–g test the concentration and incubation time of phos-tag-biotin and SA-HRP. Phos-tag-biotin recognizes the phosphate group of m6A and thus introduces SA-HRP that catalyzes the amplification of the H_2_O_2_-HQ redox system. As the binding sites became saturated, the concentration and incubation time of phos-tag-biotin and SA-HRP gradually reached stability. Therefore, the final experimental conditions were set as follows: 20 µg mL^−1^ m6A antibody with a 3 h incubation time, 1 h m6A incubation time, 10 µM phos-tag-biotin with a 1 h incubation time, and 5 U mL^−1^ SA-HRP with a 45 min incubation time.

### 2.4. Electrochemical Analysis

**Figure 5:**
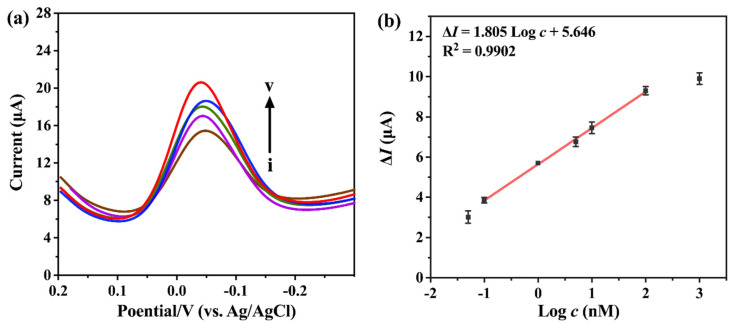
(**a**) DPVs of the assembled biosensor for different concentration of m6A in 0.1 M PB containing 1 mM hydrogen peroxide (H_2_O_2_) and 1 mM hydroquinone (HQ). Curves i–v represent the m6A concentration of 0.1, 1, 5, 10, and 100 nM. (**b**) Regression calibration plot between ΔI and logarithmic concentration of m6A. Error bars represent standard deviations of three measurements.

Under the optimum conditions, we investigated the sensitivity of the biosensors for m6A at different concentrations. Figure 5a shows the DPV plots obtained from LIG biosensors incubated in solutions of various m6A concentrations, which demonstrates that the peak current response of the H_2_O_2_-HQ redox system increases with the increase in m6A concentration. The current response has a good linear relationship with the logarithmic concentration of m6A from 0.1 nM to 100 nM (Figure 5b). The regression equation, Δ*I* = 1.805 Log *c* + 5.646 (R^2^ = 0.9902), yielded a limit of detection (LOD) of 96 pM (Δ*I* indicates the difference between methylated m6A strands and the unmethylated control A strands). The LOD was calculated by setting Log *c* equal to three times the standard deviation of 11 blanks, divided by the slope. At the same time, we compared our method with the reported methods and summarized them in SI (Table S3). At present, it seems that our method still has some limitations, and the main problem is that the detection limit is not low enough. In the future, we will focus on designing innovative electrochemical detection methods, and endeavor to make the linear range wider and the detection limit lower. Meanwhile, the detection of m6A modification on LIG has not been reported by other teams, and we will try to utilize the advantages of LIG to achieve rapid detection.

### 2.5. Specificity, Stability, and Repeatability

**Figure 6:**
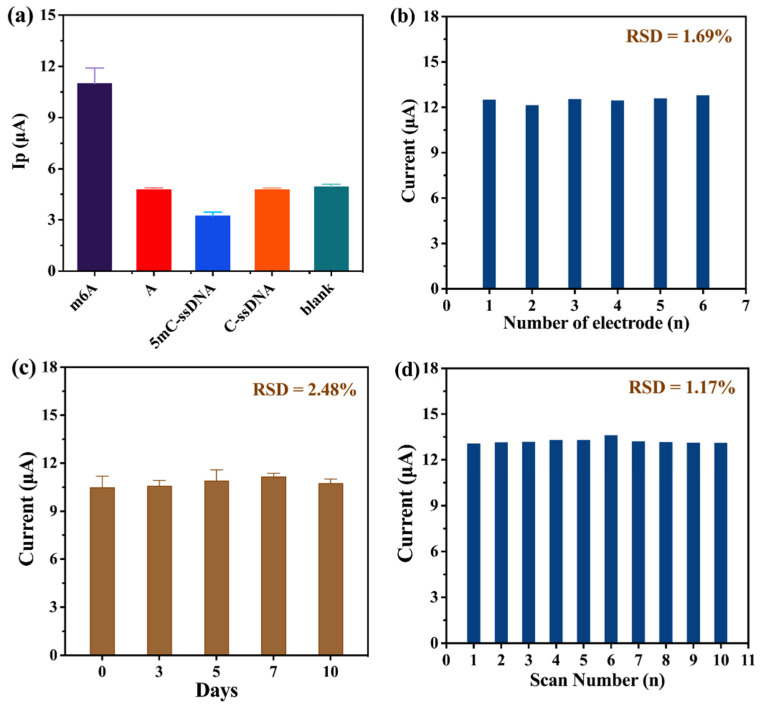
(**a**) Specificity measurement of 0.01 µM m6A, unmethylated A, methylated 5mC-ssDNA, unmethylated C-ssDNA, and blank. (**b**) Reproducibility of six independent biosensors. (**c**) Stability test of ten days. (**d**) Stability test of consecutive scanning. Error bars represent standard deviations of three measurements.

By exploring the selectivity of the developed LIG biosensor, other potential nucleic acid strands’ interferences were investigated for selectivity, including unmethylated A, methylated 5mC-ssDNA, unmethylated C-ssDNA, and a blank case without any nucleic acid strand addition. The largest peak current is observed in Figure 6a, resulting from the specific high-affinity binding of the m6A antibody to methylated m6A. However, the smaller peak currents for unmethylated A, methylated 5mC-ssDNA, unmethylated C-ssDNA, and the blank indicate that these nucleic acid chains are unable to combine with the m6A antibody, which in turn fails to produce the introduction of the subsequent molecule. This indicates a better selectivity of this method.

In addition, we investigated six independent electrodes at the same concentration of m6A (Figure 6b). The method exhibited good reproducibility, as indicated by a relative standard deviation of 1.69%. Furthermore, the storage stability at 4 °C and the consecutive scanning stability were also investigated. In Figure 6c, after storing for 0 days, 3 days, 5 days, 7 days, and 10 days, the fabricated electrodes were detected and the results showed that the peak current detected on day 10 was relatively stable compared with that on day 0, and the RSD was calculated to be 2.48%. Meanwhile, Figure 6d shows the peak current obtained from 10 consecutive scans of an LIG electrode with an RSD of 1.17%, implying that this method has better scanning stability.

### 2.6. Applications in Spiked Samples

To demonstrate our LIG electrode’s application potential in cell samples, we tested different concentrations of m6A spiked into total RNA extracted from HeLa cells. The recovery and RSD values, shown in Table 1, suggests our method holds promising potential for detecting m6A in spiked samples.

## 3. Experimental Section

### 3.1. Materials and Reagents

The highest available analytical grade reagents and chemicals were used in the experiment. Polyimide sheets (PI, 55 µm) and polyethylene terephthalate (PET) sheets were purchased from a network store (Taobao, China). Ag/AgCl paste for the reference electrode was purchased from Shanghai Julong Electronic Technology Co., Ltd. (Shanghai, China). Potassium ferrocyanide ([K_4_Fe(CN)_6_]), potassium ferricyanide ([K_3_Fe(CN)_6_]), potassium chloride (KCl), potassium nitrate (KNO_3_), hydrochloric acid (HCl), ethylene diamine tetraacetic acid (EDTA), and sodium chloride (NaCl) were purchased from Sinopharm Chemical Reagent Co., Ltd. Tris (hydroxymethyl) aminomethane (Tris) was obtained from Regal (Shanghai, China). 1,4-Dithiothreitol (DTT), bovine serum albumin (BSA), β-Cyclodextrin (β-CD), hydroquinone (HQ), and hydrogen peroxide (H_2_O_2_) were purchased from Aladdin (Shanghai, China). T4 polynucleotide kinase (T4 PNK) was purchased from Thermo Fisher Scientific (Shanghai, China) Co. Ltd. Adenosine triphosphate (ATP) was purchased from Macklin (Shanghai, China). Phos-tag-biotin was obtained from Wako (Japan). Anti-m6A-Ab was obtained from Abcam. Streptavidin-modified horseradish peroxidase (SA-HRP) was purchased from Sangon Biotech (Shanghai, China). The phosphate-buffered saline (PBS, 10×) was obtained from Solarbio (Beijing, China).

HPLC-purified oligonucleotides were obtained from TAKARA Biomedical Technology Co., Ltd. (Dalian, China) and the sequences are as follows:

m6A-RNA: 5′-GGACUGAGAGGAmCUGUCUGGGUGCCAAG-3′
Unmethylated-RNA: 5′-GGACUGAGAGGACUGUCUGGGUGCCAAG-3′

The buffers used in this study were prepared as follows: Tris-HCl buffer (pH 7.4) was used as RNA diluent, which contains 10 mM Tris, 1 mM EDTA, 1 M NaCl, and 1 mM TCEP. In addition, 0.01 M PBS was used for antibody and SA-HRP dilution; 0.01 M PB (pH 7.2) was used as an electrochemical detection buffer in the HQ-H_2_O_2_ redox system. Phos-tag-biotin buffer (pH7.5) was used for phos-tag biotin dilution, which contains 10 mM Tris-HCl, 0.1% Tween-20 (*v*/*v*), 0.4 mM Zn(NO_3_)_2_·6H_2_O, and 0.1 M NaCl. Aqueous solutions for RNA detection were prepared using DEPC water (Beyotime, Shanghai, China).

### 3.2. Apparatus

A nanophotometer (IMPLEN, Munich, Germany) was used for m6A concentration quantification. A field-emission scanning electron microscope (Zeiss Sigma, Oxford, UK) was used for LIG and β-CD/LIG morphology observation. All the electrochemical measurements were performed on the CHI 660D electrochemical workstation (Shanghai Chenhua Instruments Co., Ltd., Shanghai, China). Electrochemical impedance spectroscopy (EIS) measurements were performed using the VersaSTAT 3F electrochemical workstation (Ametek, Berwyn, PA, USA). Laser-induced graphene was produced by a laser engraving machine (Shanghai Diaotu Industrial Co., Ltd., Shanghai, China). An X-ray photoelectron spectrometer (ESCALAB250Xi, Thermo Fisher Scientific, Waltham, MA, USA) was used for surface elemental analysis of LIG and β-CD/LIG.

### 3.3. Fabrication of LIG and β-CD/LIG

Pre-designed patterned three-electrode integrated LIG electrodes were prepared in one step on PI film using the laser direct writing technique. The laser parameters were 30% power, 20% speed, and a 25 mm focusing height. Briefly, PI film was uniformly pasted on a PET sheet and cleaned with ultrapure water and 75% ethanol, respectively. Then, the PI sheet was placed under a semiconductor laser source for further engraving. Finally, the working area was secured with PVC tape and Ag/AgCl paste was used as a reference electrode after being dried in an oven at 60 °C. The electropolymerization of β-CD on the working electrode of LIG was conducted in 6 mM aqueous solution of β-CD using the amperometry method with the following parameters: a potential of −2.0 V and a deposition time of 300 s. The reference electrode we used was Ag/AgCl coated on an LIG electrode. To determine potential, we referred to the cyclic voltammetry polymerization potential range used within the two papers [52,53].

### 3.4. Assembly of Molecules on LIG

Since the ends of the model strands we used for experiments were hydroxyl-modified, phosphorylation-modified m6A was obtained by phosphorylating the 5′ end of the m6A strand according to the literature with some modifications [54]. A total of 34.5 µL of DEPC water, 5 µL of 100 µM m6A, 5 µL of 10× T4 PNK buffer, 2.5 µL of 0.1 M DTT, 2 µL of 10 U mL^−1^ T4 PNK, and 1 µL of 25 mM ATP were mixed and incubated at 37 °C for 12 h. The reaction was purified using the Oligo Clean and Concentrator Kit, quantified using Nanodrop, and kept at −20 °C for further use.

**Scheme 1:**
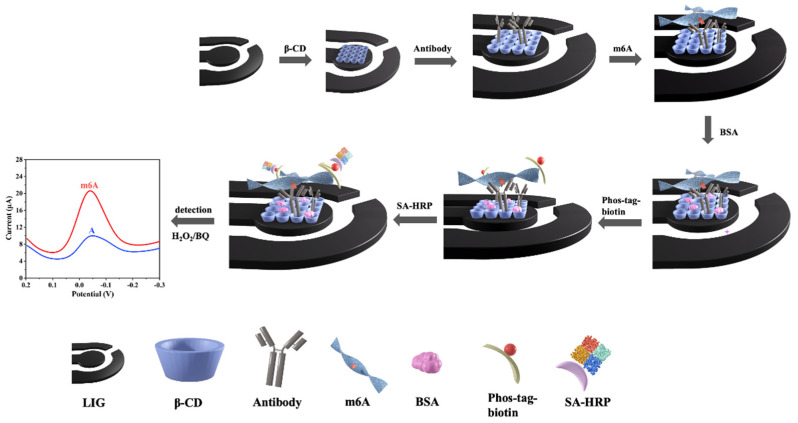
Schematic diagram of the assembly process on laser−induced graphene (LIG).

The assembly process on LIG is shown in Scheme 1. After the β-CD/LIG was fabricated, 2 µL of 20 µg mL^−1^ anti-m6A antibody was first dropped onto the working electrode of β-CD/LIG and incubated for 3 h. Anti-m6A antibody was then immobilized on β-CD/LIG via noncovalent bonding, according to the method in the published literature [52,53]. Then, 2 µL of different concentrations of m6A were added dropwise to the electrode for 1 h to achieve antigen–antibody specific recognition. The electrode was blocked using 1% BSA to reduce the specific adsorption. Next, 3 µL of 10 µM phos-tag-biotin was used to recognize the phosphate group on m6A, thus introducing biotin. Finally, 3 µL of 5 U mL^−1^ SA-HRP was added to complete the binding of biotin and streptavidin, and the ability of HRP to catalyze the HQ-H_2_O_2_ redox system caused the current signal to increase. As the concentration of methylated chains increased, the current signal became higher, allowing for quantification of m6A. After each modification step, the electrode was rinsed with DEPC water to remove unreacted solution and was dried at room temperature. In addition, all of the above steps were performed at 37 °C.

### 3.5. Electrochemical Detection of m6A

Differential pulse voltammetry (DPV) was used for the electrochemical detection of m6A-RNA in 0.1 M PB solution (pH 7.4) containing 1 mM HQ and 1 mM H_2_O_2_. The following parameters were used: initial potential at 0.2 V, final potential at −0.3 V, amplitude at 0.05 V, pulse width of 0.05 s, sample width of 0.01667 s, pulse period of 0.2 s, quiet time of 2 s, and sensitivity of 1 × 10^−5^.

### 3.6. Total RNA Isolation and Purification

Total RNA (tRNA) was extracted from human cervical carcinoma cell lines (HeLa cells) using the TRIzol method. Spiked samples contained different concentrations of the model chains and the same concentration of the cellular extracted chains.

## 4. Conclusions

In summary, we developed a highly sensitive electrochemical platform utilizing β-CD-modified 3D porous LIG electrodes. The step-by-step modification of the LIG electrode surface, including antibody binding to β-CD through non-covalent interactions, specific m6A recognition, BSA blocking of nonspecific sites, m6A phosphate group recognition by phos-tag-biotin, and subsequent SA-HRP introduction, enabled successful electrochemical m6A detection. Demonstrating excellent selectivity, repeatability, and sensitivity, this method effectively detected m6A in spiked tRNA samples with satisfactory recoveries. Furthermore, LIG offers a straightforward, cost-effective, easily modifiable, and eco-friendly production process, making it suitable for industrial scaling. The rapid detection capability of this method holds significant promise for clinical m6A POCT, crucial for early diseases diagnosis. Nevertheless, further optimization to enhance sensitivity and achieve lower limits of detection is required for its application in detecting m6A in authentic biological samples.

## Figures and Tables

**Table 1:** Recovery of m6A in spiked samples.

Added (ng/µL)	Acquired (ng/µL)	Recovery (%)	RSD (%, N = 3)
0.05	0.05 ± 0.002	101.59	4.87
0.25	0.25 ± 0.025	100.42	9.84
0.5	0.52 ± 0.038	104.01	7.23
0.75	0.78 ± 0.074	104.08	9.51

## Data Availability

The raw data supporting the conclusions of this article will be made available by the authors on request.

## Supplementary Materials

The following supporting information can be downloaded at https://www.mdpi.com/article/10.3390/molecules29194718/s1, Table S1: Atomic percentage of C, N, and O species in LIG and β-CD/LIG samples; Table S2: The effective surface area (A); Table S3: Electrochemical strategies for m6A detection.
