# Supplementary material for: β-Cyclodextrin-Modified Laser-Induced Graphene Electrode for Detection of N6-Methyladenosine in RNA
Source: Molecules. 2024 Oct 5;29(19):4718. doi: 10.3390/molecules29194718 (PMC11478181; doi:10.3390/molecules29194718)
Supplement: Supplementary file 1 [file molecules-29-04718-s001.zip › molecules-3236817-supplementary.pdf]

Supporting Information

β-Cyclodextrin Modified Laser-Induced Graphene Electrode for Detection of N6-methyladenosine in RNA

Jingyi Guo [a], Mei Zhao [a], Xia Kuang [a], Zilin Chen [a], Fang Wang *[a]

[a] *School of Pharmaceutical Sciences, Key Laboratory of Combinatorial Biosynthesis and Drug Discovery (MOE), Wuhan University, Wuhan, 430071, China. Email: fwang@whu.edu.cn*

Corresponding author:

Prof. Fang Wang

Tel: 86-27-68759829, fax: 86-27-68759850.

E-mail address: fwang@whu.edu.cn

Contents：

1. Atomic percentage of C, N, and O species in LIG and β-CD/LIG samples.

2. Table of the effective surface area (A).

3. Electrochemical strategies for m6A detection.

Table S1 Atomic percentage of C, N, and O species in LIG and β-CD/LIG samples.

Sample	C%	N%	O%
LIG	88.32	3.89	7.79
β-CD/LIG	72.00	2.18	21.97

Table S2 The effective surface area (A).

Modification step	Surface area (cm^2)
β-CD /LIG	0.191
Ab/β-CD /LIG	0.189
m6A/Ab/β-CD /LIG	0.163
BSA/m6A/Ab/β-CD /LIG	0.148
phos-tag-biotin/BSA/m6A/Ab/β-CD /LIG	0.132
SA-HRP//phos-tag-biotin/BSA/m6A/Ab/β-CD /LIG	0.107

The experimental parameters used in Randles-Sevcik equation:

$$I_p = 2.69 \times 10^5 \, A \, n^{3/2} \, {D_0}^{1/2} \, C_0 \, v^{1/2}$$

n = 1, D_0 = 6.73 × 10^{-6} cm^2/s, C_0 = 2.5 mM, v = 100 mV/s

Table S3. Electrochemical strategies for m6A detection.

Electrode	Signal amplification strategy	Linear range	LOD	Refs
Au electrode	Immune competition of m6A-RNA and m6A-DNA with RNase helped signal amplification	0.05–200 nM	16 pM	[1]
graphene-AuNPs /GCE	Ag@SiO_2 as signal amplification label	0.2-500 nM	0.078 nM	[2]
AuNPs/GCE	HRP-IgG recognizes anti-m6A-antibody and catalyzes H_2O_2-HQ redox system	0.0001-10 nM	0.094 pM	[3]
AuNPs/GCE	Decreased signal of $[Fe(CN)_6]^{3-}$/$[Fe(CN)_6]^{4-}$ caused by antibody recognizing methylated RNA	0.01-10 nM	2.57 pM	[4]
Au electrode	Competition of m6A-RNA and m6A-DNA-PtCo and PtCo catalyzing H_2O_2 reduction	0.005-100nM	2.1 pM	[5]
AuNPs/Au	RNA ligase helping hybridization and HRP-IgG-AuNPs catalyzing H_2O_2-HQ redox system	10 fM-10 nM	3.35 fM	[6]
β-CD/LIG	Phos-tag-biotin binding SA-HRP and catalyzing H_2O_2-HQ redox system	0.1–100 nM	0.096 nM	This work

References

[1] T. Dai, Q. L. Pu, Y. C. Guo, C. Zuo, S. L. Bai, Y. J. Yang, D. Yin, Y. Li, S. C. Sheng, Y. Y. Tao, J. Fang, W. Yu and G. M. Xie, Analogous Modified DNA Probe and Immune Competition Method-Based Electrochemical Biosensor for RNA Modification, *Biosens Bioelectron*. 114 (2018) 72-77. 10.1016/j.bios.2018.05.018.

[2] H. S. Yin, H. Y. Wang, W. J. Jiang, Y. L. Zhou and S. Y. Ai, Electrochemical Immunosensor for N6-Methyladenosine Detection in Human Cell Lines Based on Biotin-Streptavidin System and Silver-SiO(2) Signal Amplification, *Biosens Bioelectron*. 90 (2017) 494-500. 10.1016/j.bios.2016.10.066.

[3] H. M. Yang, Y. F. Wang, J. Tang, F. Wang and Z. L. Chen, End-Labeling-Based Electrochemical Strategy for Detection of Adenine Methylation in Nucleic Acid by Differential Pulse Voltammetry, *Microchim Acta*. 188 (2021) 250. 10.1007/s00604-021-04898-8.

[4] H. S. Yin, Y. L. Zhou, Z. Q. Yang, Y. L. Guo, X. X. Wang, S. Y. Ai and X. S. Zhang, Electrochemical Immunosensor for N6-Methyladenosine RNA Modification Detection, *Sensor Actuat B-Chem*. 221 (2015) 1-6. 10.1016/j.snb.2015.06.045.

[5] X. Y. Ou, Q. L. Pu, S. C. Sheng, T. Dai, D. Gou, W. Yu, T. Y. Yang, L. Dai, Y. J. Yang and G. M. Xie, Electrochemical Competitive Immunodetection of Messenger RNA Modified with N6-Methyladenosine by Using DNA-Modified Mesoporous PtCo Nanospheres, *Microchim Acta*. 187 (2020) 31. 10.1007/s00604-019-4010-8.

[6] Z. Li, B. C. Li, H. S. Yin, Q. H. Zhang, H. Y. Wang, H. Fan and S. Y. Ai, Electrochemical immunosensor based on hairpin DNA probe for specific detection of N6-methyladenosine RNA, *J Electroanal Chem*. 804 (2017) 192-198. 10.1016/j.jelechem.2017.09.055.
